# Health worker knowledge of Integrated Disease Surveillance and Response standard case definitions: a cross-sectional survey at rural health facilities in Kenya

**DOI:** 10.1186/s12889-018-5028-2

**Published:** 2018-01-17

**Authors:** Mitsuru Toda, Dejan Zurovac, Ian Njeru, David Kareko, Matilu Mwau, Kouichi Morita

**Affiliations:** 1 Nagasaki University Institute of Tropical Medicine, KEMRI-NUITM, Kenyatta Hospital Grounds, Nairobi, Kenya; 2 Oxford University, Oxford, UK; 3 Kenya Medical Research Institute-Wellcome Trust Research Programme, Nairobi, Kenya; 4 Kenya Ministry of Health Disease Surveillance and Response Unit, Nairobi, Kenya; 5 Kenya Medical Research Institute, Nairobi, Kenya; 6 Nagasaki University Institute of Tropical Medicine, Nagasaki, Japan

**Keywords:** Case definitions, Knowledge, Health workers, Disease surveillance, Kenya

## Abstract

**Background:**

The correct knowledge of standard case definition is necessary for frontline health workers to diagnose suspected diseases across Africa. However, surveillance evaluations commonly assume this prerequisite. This study assessed the knowledge of case definitions for health workers and their supervisors for disease surveillance activities in rural Kenya.

**Methods:**

A cross-sectional survey including 131 health workers and their 11 supervisors was undertaken in two counties in Kenya. Descriptive analysis was conducted to classify the correctness of knowledge into four categories for three tracer diseases (dysentery, measles, and dengue). We conducted a univariate and multivariable logistic regression analyses to explore factors influencing knowledge of the case definition for dysentery.

**Results:**

Among supervisors, 81.8% knew the correct definition for dysentery, 27.3% for measles, and no correct responses were provided for dengue. Correct knowledge was observed for 50.4% of the health workers for dysentery, only 12.2% for measles, and none for dengue. Of 10 examined factors, the following were significantly associated with health workers’ correct knowledge of the case definition for dysentery: health workers’ cadre (aOR 2.71; 95% CI 1.20–6.12; *p* = 0.017), and display of case definition poster (aOR 2.24; 95% CI 1.01–4.98; *p* = 0.048). Health workers’ exposure to the surveillance refresher training, supportive supervision and guidelines were not significantly associated with the knowledge.

**Conclusion:**

The correct knowledge of standard case definitions was sub-optimal among health workers and their supervisors, which is likely to impact the reliability of routine surveillance reports generated from health facilities.

## Background

The first step in disease surveillance for frontline health workers is to diagnose and identify cases at health facilities. Rural health facilities in sub-Saharan Africa lack capacities to diagnose diseases through laboratory confirmations and health workers rely on clinical signs and symptoms to diagnose suspected cases of priority diseases. Validated clinical standard case definitions help standardize diagnosis of suspected cases across health workers within and across nations, and it is crucial for implementing the International Health Regulations 2005 [1–3]. In addition, standard case definitions are important for managers at all levels to monitor epidemic trends across health facilities, investigate epidemic alerts, respond to potential outbreaks, and plan activities related to disease surveillance and control.

In Kenya as in other African countries, the World Health Organization regional office for Africa (WHO-Afro) implemented the Integrated Disease Surveillance and Response (IDSR) strategy [4, 5], which promotes the use of IDSR standard case definitions which are stipulated in technical guidelines, promoted through job aids, and included in the in-service training programs. In IDSR evaluation literature, processes of disease surveillance and activities supporting implementation have commonly been examined [6–17]. However, the knowledge of IDSR standard case definitions by health workers and their supervisors have been assumed and no study has examined the awareness and knowledge of case definitions as stipulated in the guidelines [18, 19]. We therefore conducted a cross-sectional study among frontline health workers and their disease surveillance supervisors in rural Kenya to assess their knowledge of IDSR standard case definitions.

## Methods

A cross-sectional survey was conducted as part of the mSOS (mobile Short-message-service based disease Outbreak alert Systems) trial [20], which measured the effectiveness of an intervention using text-message disease outbreak alert system. The study took place at rural health facilities and sub-county management offices in Busia and Kajiado counties in Kenya.

### Description of study area

Busia County is by the Victoria Lake basin and borders Uganda, and Kajiado County borders Tanzania. In the two counties, there are 143 functional health facilities belonging to 11 sub-counties. All health facilities are expected to report priority diseases through IDSR strategy to disease surveillance coordinators at the sub-county level. IDSR strategy was introduced to health workers in the two counties in 2005. Health facility in-charges, or IDSR focal persons at each health facility are required to send routine paper-based reports on priority diseases to sub-county office on immediate and weekly basis. Sub-county level disease surveillance coordinators are expected to monitor disease trends, respond to alerts of disease surveillance activities, and provide support supervision to the health facilities. Use of standard case definition has been promoted through the IDSR strategy in the area for over a decade. In addition, in September and October 2013, health facility in-charges in the study area attended an IDSR refresher training organized by the Ministry of Health. During the 1-day training, IDSR case definitions and reporting requirements were highlighted. Each participant was given the IDSR technical guidelines for use at their health facility, and participants were expected to have refreshed their knowledge on IDSR standard case definitions.

### Participants and data collection

Of 143 health facilities, 12 health facilities were excluded from the study because the facilities were closed on the day of the survey. Participants included 131 health facility in-charges and 11 sub-county disease surveillance coordinators who were interviewed by the study team in April 2014, which was 6 months after the IDSR refresher training took place in the study area. Open-ended questionnaires were self-administered by health facility in-charge and their sub-county supervisors at their respective workstations on their knowledge of IDSR standard case definitions. The questionnaires were pre-tested in Nairobi prior to study collection. National level Ministry of Health staff members at the Disease Surveillance and Response Unit, who are well experienced and trained in IDSR strategy, supervised the collection of data at each study facility. The responses were recorded in English because it is the language for pre- and in- service trainings in Kenya.

### Selection of tracer diseases and study definition

Three tracer diseases, dysentery, measles and dengue, were selected for analysis based on the epidemiological interest [21] and the complexity of the IDSR standard case definitions (Table 1). Dysentery was selected because the disease occurs frequently [22–24] and has only 2 simple key components in the case definition. Measles was selected because there are sporadic outbreaks in the country [25, 26] and has slightly more complex case definition than dysentery containing 5 key components. Dengue was selected because the disease is reported frequently in the research setting [27–34] and has a complex case definition containing 8 key components. All of the three tracer diseases require weekly reporting according to the national IDSR guidelines, while measles and dengue are also required to be reported immediately through the case-based reports as suspected cases are detected. Table 1 presents study definitions for each of the three tracer diseases by four categorization of the correctness: correct, partially correct, incorrect, and “do not know”. Responses were classified as correct when all key components were stated without other signs or symptoms. Partial correctness was defined as mentioning all of the key components, or mentioning some of key components with additional disease related symptoms. Incorrect responses were defined as mentioning only one or none of the key components. Finally, the last category “do not know” included answers where respondents did not mention any signs or symptoms.

**Table 1:** Standard case definitions according to IDSR national guidelines and study classifications

	Standard case definitions (suspected case) in the IDSR technical guidelines	Study definitions based on key words	
Dysentery (Shigella)	A person with diarrhoea with visible blood in stool	Correct	Diarrhea and blood
		Partially correct	Diarrhea, blood, and other related symptoms^1^
		Incorrect	No mention of diarrhea or blood
		Do not know	No mention of signs and symptoms
Measles	Any person with fever and maculopapular (non-vesicular) generalized rash and any one of the following: cough, coryza (irritation and inflammation of the mucosal membrane of the nose), conjunctivitis (red eyes), any person in whom a clinician suspects measles	Correct	Fever, rash, cough, coryza, and conjunctivitis
		Partially correct	Fever, rash, and at least one of the following symptoms: cough, coryza, and conjunctivitis; with or without related symptoms
		Incorrect	No mention of rash or fever
		Do not know	No mention of signs and symptoms
Dengue fever	Any person with acute febrile illness of 2–7 days duration with 2 or more of the following: headache, retro-orbital pain (pain behind the eyes), myalgia (muscle pain), arthralgia (joint pain), rash, haemorrhagic manifestations, leukopenia (low white blood cells)	Correct	Fever, headache, retro-orbital pain, myalgia, arthralgia, rash, hemorrhagic manifestations, and leukopenia
		Partially correct	Fever and at least two of the following symptoms: headache, retro-orbital pain, myalgia, arthralgia, rash, hemorrhagic manifestations, and leukopenia
		Incorrect	No mention of fever; or mention of fever with less than two of the following symptoms: headache, retro-orbital pain, myalgia, arthralgia, rash, hemorrhagic manifestations, and leukopenia
		Do not know	No mention of signs and symptoms

^1^Other related symptoms include: abdominal pain/cramps, fever, and frequency of diarrhea in a day

### Data analysis

Data were double entered using Base (LibreOffice 5, The Document Foundation, Berlin, Germany). Study investigators verified data manually comparing paper based questionnaires. During the coding process, we classified key words of signs and symptoms according to our study definitions for the three tracer diseases. Certain key words were included as synonyms, such as diarrhea and watery stool; eye discharge, red eyes and conjunctivitis; funny nose and coryza; pain in the eyes and retro-orbital pain; general pain, muscle pain and myalgia; joint pain and arthralgia; and low blood cells and leukopenia.

Descriptive analysis of IDSR standard case definition was conducted to categorize the correctness of knowledge of three tracer diseases. To explore factors influencing the knowledge of IDSR standard case definition, we focused on dysentery because the variability of correct responses was observed only for this tracer disease. We first conducted a univariate analysis and estimated odds ratios (OR), *p*-values, and 95% confidence intervals (CIs) for the association between 10 broad factors and correct dysentery knowledge outcome. The 10 factors included health worker and health facility characteristics. In specific, location and size of facility (dispensaries: government funded health facility that provides primary healthcare services, maternity homes: private or NGO funded health facility that cares for pregnant women, medical clinics: private health facility that provides primary healthcare services, and nursing homes: private or NGO funded health facility that cares for the elderly), availability of IDSR focal person at facility, age, gender, qualification (nurses: licensed medical practitioner with 3–4 years of pre-service training, clinical officer: licensed medical practitioner with 3–4 years of pre-service training, and medical doctor: licensed medical practitioner with 6 years of pre-service training), and exposure to IDSR interventions by health workers. Factors with *p*-value <0.2 were then entered into multivariable model. We also explored two-way interaction terms of 4 variables related to disease surveillance interventions. The variables were: exposure to training, availability of guidelines, availability of poster, and support supervision. None of these 4 factors in the interaction terms were statistically significant at 0.05 level and we did not include the interaction terms in the model. Analysis was performed using Stata version 14 (StataCorp, College Station, TX, USA). Hypothesis testing and CI estimation were completed with alpha level of 0.05.

## Results

### Characteristics of health workers and their supervisors

Of 11 supervisors interviewed, 7 were from Busia (64.6%). All were public health officers. Nearly all disease surveillance coordinators were male (90.9%) and the median age of the coordinators was 44 years [IQR 38–46]. Of 131 respondents (Table 2), nearly 40 % were from Busia (36.6%). Most commonly, they worked at dispensaries (70.2%). Nearly two-thirds (64.1%) worked at government-owned public facilities, and over two-thirds (69.5%) worked at health facilities with an IDSR focal person. Half of the respondents were female (51.9%) and the median age of the respondents was 36 years [IQR 30–48]. The majority were nurses (70.2%). Around 20 % were clinical officers, who are clinicians with 3 years of pre-service training (20.6%). Regarding the surveillance related interventions, over two-thirds (68.7%) had IDSR case definition poster displayed, observed by study data collectors. Most of the respondents (74.1%) had received at least one support supervision visit on surveillance related matters in the past 6 months. Finally, only 15.3% had access to IDSR technical guidelines at their facility, while the majority (61.1%) attended the IDSR refresher training conducted by the study team 6 months earlier (Table 2).

**Table 2:** General characteristics of health facility workers

*N* = 131	n (%)
County	
Busia	48 (36.6)
Kajiado	83 (63.4)
Health facility level	
Hospital	10 (7.6)
Health centre	29 (22.1)
Dispensary^a^	92 (70.2)
Health facility ownership	
Public	84 (64.1)
Private	28 (21.4)
NGO/FBO	19 (14.5)
Working at health facility with IDSR focal person	91 (69.5)
Age; median [IQR]^b^	36 [30–48]
Gender	
Female	68 (51.9)
Male	63 (48.1)
Qualification	
Nurse	92 (70.2)
Clinical officer	27 (20.6)
Medical doctor	2 (1.5)
Other cadre^c^	10 (7.6)
Exposure to disease surveillance interventions	
Availability of poster	90 (68.7)
Availability of guidelines	20 (15.3)
Received support supervision in the last 6 months	97 (74.1)
Attended training in 2013	80 (61.1)

^a^Includes: 18 medical clinics, 3 maternity homes, and 6 nursing homes

^b^Excludes 2 health worker with missing age information

^c^Other includes: 1 Community Health Extension Worker, 1 health records officer, 1 public health officer, 1 Prevention of Mother to Child Transmission worker, and 6 laboratory technician/technologist

Abbreviations

*CO* clinical officer, *FBO* faith-based organization, *IDSR* integrated disease surveillance and response, *IQR* interquartile range, *MD* medical doctor, *NGO* non-governmental organization

### Knowledge of IDSR case definitions

Table 3 describes the knowledge of three tracer diseases by health workers and their supervisors. Of 11 supervisors, 81.8% knew the correct definition for dysentery, 27.3% for measles, and no correct responses were provided for dengue. For measles, 36.4% responded partially correctly and 27.3% incorrectly. For dengue, only one supervisor provided partially correct response (9.1%), nearly half responded incorrectly (45.5%), and the remaining 45.5% did not know the definition (Table 3).

**Table 3:** Respondents’ knowledge of IDSR standard case definitions

	Dysentery	Measles	Dengue
	n (%)	n (%)	n (%)
Sub-county disease surveillance coordinator *N* = 11			
Correct	9 (81.8)	3 (27.3)	0
Partially correct	1 (9.1)	4 (36.4)	1 (9.1)
Incorrect	1 (9.1)	3 (27.3)	5 (45.5)
Do not know	0	1 (9.1)	5 (45.5)
Health facility in-charges *N* = 131			
Correct	66 (50.4)	16 (12.2)	0
Partially correct	23 (17.6)	54 (41.2)	5 (3.8)
Incorrect	22 (16.8)	36 (27.5)	41 (31.3)
Do not know	20 (15.3)	25 (19.1)	85 (64.9)

Health workers displayed similar patterns as their supervisors across the three tracer diseases. Correct knowledge was observed for 50.4% of the health workers for dysentery, for only 12.2% for measles, and none of the health workers provided correct case definition for dengue. For dysentery, 17.6% responded partially correctly where bloody diarrhea was mentioned but also additional dysentery related signs and symptoms such as pain or cramps (11/23) and fever (3/23). Furthermore, twenty-two health workers (16.8%) provided incorrect case definitions by either omitting diarrhea (16/22) or blood (6/22). Finally, the remaining 20 health workers (15.3%) did not know the definition for dysentery (Table 3).

For measles, less than half of health workers responded partially correctly (41.2%) where health workers were able to mention fever and rash, but not all of the additional three components (cough, coryza, and conjunctivitis). Most commonly omitted symptoms were cough (37/54), followed by coryza (29/54), and conjunctivitis (12/54). Over one-quarter of health workers responded incorrectly (27.5%) by omitting fever (29/36) followed by rash (12/36). The remaining 19.1% of the health workers did not know the case definition for measles (Table 3).

Nearly all (96.2%) health workers either incorrectly stated or did not know the case definition for dengue. Among the incorrect responses, three did not mention fever (3/41), while other dengue criteria were rarely mentioned: headache (9/41), arthralgia (8/41), hemorrhagic manifestations (6/41), retro-orbital pain (2/41), and rash (2/41), myalgia (0/41), and leukopenia (0/41).

### Predictors influencing knowledge of dysentery

We examined 10 factors that may influence the health facility in-charges’ knowledge of dysentery. The following variables did not meet entrance criteria (*p* < 0.2) for the multivariable analysis: county (OR 1.11; 95% CI 0.55–2.27; *p* = 0.767), smaller facilities (OR 1.47; 95% CI 0.69–3.13, *p* = 0.313), age (OR 1.23; 95% CI 0.62–2.47; *p* = 0.553), gender (OR 1.09; 95% CI 0.55–2.17; *p* = 0.796), training (OR 1.41; 95% CI 0.70–2.86; *p* = 0.335), presence of IDSR technical guidelines (OR 0.61; 95% CI 0.23–1.61; *p* = 0.316), and support supervision (OR 0.87; 95% CI 0.40–1.90; *p* = 0.729) (Table 4). The following factors were significantly associated with correct dysentery IDSR standard case definition knowledge in the multivariable analysis: health workers’ cadre (aOR 2.71; 95% CI 1.20–6.12; *p* = 0.017), and display of case definition poster (aOR 2.24; 95% CI 1.01–4.98; *p* = 0.048). Although meeting the inclusion criteria for multivariable model, the presence of IDSR focal person at the health facility (aOR 1.83; 95% CI 0.82–4.09; *p* = 0.139) was not significantly associated with correct dysentery knowledge (Table 4).

**Table 4:** Predictors influencing knowledge of dysentery: univariate and multivariable regression results

	N	n (%)	OR [95% CI]	*p*-value	aOR [95% CI]	*p*-value
General characteristics						
County						
Busia	48	25 (52.1)	1.11 [0.55–2.27]	0.767	–	–
Kajiado	83	41 (49.4)	1.0 (Ref)		–	
Size of facility						
Smaller^a^	92	49 (53.3)	1.47 [0.69–3.13]	0.313	–	–
Larger^b^	39	17 (43.6)	1.0 (Ref)		–	
IDSR focal person at facility						
Available	91	50 (55.0)	1.83 [0.86–3.89]	0.117	1.83 [0.82–4.09]	0.139
Not available	40	16 (40.0)	1.0 (Ref)		1.0 (Ref)	
Age^c^						
35+ years old	71	38 (53.5)	1.23 [0.62–2.47]	0.553	–	–
20–34 years old	58	28 (48.3)	1.0 (Ref)		–	
Gender						
Female	68	35 (51.5)	1.09 [0.55–2.17]	0.796	–	–
Male	63	31 (49.2)	1.0 (Ref)		–	
Qualification						
Nurse	92	53 (57.6)	2.72 [1.24–5.95]	0.012	2.71 [1.20–6.12]	0.017
MD/CO/other^d^	39	13 (33.3)	1.0 (Ref)		1.0 (Ref)	
Exposure to IDSR interventions						
Training						
Attended	80	43 (53.8)	1.41 [0.70–2.86]	0.335	–	–
Did not attend	51	23 (45.1)	1.0 (Ref)		–	
Case definition poster						
Displayed	90	52 (57.8)	2.64 [1.22–5.70]	0.013	2.24 [1.01–4.98]	0.048
Not displayed	41	14 (34.2)	1.0 (Ref)		1.0 (Ref)	
Technical guidelines						
Available	20	8 (40.0)	0.61 [0.23–1.61]	0.316	–	–
Not available	111	58 (52.3)	1.0 (Ref)		–	
Supervision in past 6 months						
Received	97	48 (49.5)	0.87 [0.40–1.90]	0.729	–	–
Not received	34	18 (52.9)	1.0 (Ref)		–	

^a^Includes 65 dispensaries, 3 maternity homes, 18 medical clinics, and 6 nursing homes

^b^Includes 29 health centers and 4 hospitals

^c^The denominator excludes 2 health worker with missing age information

^d^Other includes: 1 Community Health Extension Worker, 1 health records officer, 1 public health officer, 1 Prevention of Mother to Child Transmission worker, and 6 laboratory technician/technologist

Abbreviations

*aOR* adjusted odds ratio, *CO* clinical officer, *IDSR* integrated disease surveillance and response, *MD* medical doctor

## Discussion

Correct knowledge of standard case definitions is an important first step for successful implementation of any disease surveillance strategy. Our study, one of the first that examined and quantified the knowledge of IDSR standard case definitions in sub-Saharan Africa, revealed alarmingly low levels of knowledge in Kenya. Patient registers at health facilities are used as source document for evaluating disease surveillance reporting practices and health systems in general [35], and our study findings imply that the validity of the source documents may be overlooked. First, we found that knowledge of dengue is practically non-existent among health workers and their disease surveillance supervisors. We speculate that the relatively complex case definitions, lack of routine laboratory diagnosis to confirm dengue in rural health facilities, similarities with other mosquito-borne diseases such as malaria, and the possibility of perceived non-lethal nature of the disease may contribute to low knowledge. The findings are however not unique to our study sites since suboptimal knowledge of dengue has also been reported among health workers in high-income countries [36]. Similarly to dengue but even more surprising finding was found with respect to measles. Despite simple case definitions, only a quarter of supervisors and just one in ten health workers could state five clinical criteria, which have been promoted in Kenya over a decade. It should also be acknowledged that surveillance guidelines in Kenya include an ambiguous and unnecessary wording, “any person in whom a clinician suspects measles”, which undermines the validity of promoted case definitions and itself precludes standardization. Similar sentiments were expressed in an evaluation conducted in South Sudan [37]. The most unexpected finding was observed on dysentery where only half of the health workers were able to correctly state the simple criteria of “bloody diarrhea.” Health workers responses of other dysentery related symptoms such as “abdominal cramps and pains” may suggest the health workers are knowledgeable of the text book clinical features [38], but disregarding the IDSR guidelines.

Our predictor analysis examining factors for correct knowledge added further light on what may influence knowledge of dysentery. First, we found that nurses are significantly more knowledgeable compared to other cadres of health workers. One of the possible explanations is that health workers with greater pre-service clinical training are more likely to overrule guidelines, which has been shown in previous studies [39, 40]. Second, the presence of a simple job aid in the form of a case definition poster at a health facility was significantly associated with better knowledge. Reminders are shown to be effective intervention in other studies [41], and policy makers should prioritize such seemingly simple intervention. In addition, this study was conducted in the context of evaluating a text message intervention to enhance disease surveillance reporting [20] and reminder interventions through such channels but targeting health workers should also be explored in the future. Finally, exposure to either refresher training or supervision was not associated with greater knowledge of case definition. We do not think that these interventions are ineffective per se, but perhaps the sub-optimal quality of the training and supervision intervention may have contributed to the analysis results. The study did not have a component to measure the quality of training or resources to follow-up supervision mechanisms after training, but we speculate that these factors may have contributed to low knowledge levels. Moreover, the low knowledge levels of disease surveillance supervisors found in this study further elevates the levels of challenges faced in the field to promote adherence to IDSR standard case definitions.

Several study limitations should be acknowledged. First, the knowledge levels were self-reported by health workers and the study did not examine whether the health workers’ knowledge was translated into practice. We also did not examine the duration of professional experience that may have influenced the level of knowledge. Knowledge is however a prerequisite for correct practice and it is unlikely that health workers would adhere to guidelines. We also cannot confidently assume uniformity in diagnosis of suspected cases when knowledge of the standard case definition is not present. Second, we limited the analysis to three tracer diseases and applied a rather relaxed approach to interpreting the definitions. Given the low knowledge levels found overall in the study, stricter case definitions would have however yielded even lower knowledge results, and likely without changes to the interpretation of the findings. Third, the small sample size used in this study may have precluded showing significant associations when associations were truly present, and results should be interpreted with caution. Finally, while the study included a number of health workers from two areas in Kenya, the results are not nationally representative and cannot be generalized across the country.

## Conclusion

Overall, the IDSR case definition knowledge needs to be further improved among both health workers and their supervisors, and the findings are likely to impact the reliability of routine surveillance reports generated from health facilities. The findings also suggest that simple intervention such as reminders are more effective than ad hoc trainings and routine supervision to ensure uniformed knowledge of case definitions. Further studies on examining effective methods of enhancing knowledge of IDSR standard case definitions are justified.
